# Using quantitative real-time PCR to detect chimeras in transgenic tobacco and apricot and to monitor their dissociation

**DOI:** 10.1186/1472-6750-10-53

**Published:** 2010-07-16

**Authors:** Mohamed Faize, Lydia Faize, Lorenzo Burgos

**Affiliations:** 1 Grupo de Biotecnología, Departamento de Mejora de Frutales. CEBAS-CSIC, P.O. Box 164, 30.100 Murcia, Spain; 2 Laboratoire de Biologie et Biotechnologie Végétales. Faculté des Sciences, Université Chouaib Doukkali, 24000 El Jadida, Morocco

## Abstract

**Background:**

The routine generation of transgenic plants involves analysis of transgene integration into the host genome by means of Southern blotting. However, this technique cannot distinguish between uniformly transformed tissues and the presence of a mixture of transgenic and non-transgenic cells in the same tissue. On the other hand, the use of reporter genes often fails to accurately detect chimerical tissues because their expression can be affected by several factors, including gene silencing and plant development. So, new approaches based on the quantification of the amount of the transgene are needed urgently.

**Results:**

We show here that chimeras are a very frequent phenomenon observed after regenerating transgenic plants. Spatial and temporal analyses of transformed tobacco and apricot plants with a quantitative, real-time PCR amplification of the neomycin phosphotransferase (*npt*II) transgene as well as of an internal control (β-*actin*), used to normalise the amount of target DNA at each reaction, allowed detection of chimeras at unexpected rates. The amount of the *npt*II transgene differed greatly along with the sub-cultivation period of these plants and was dependent on the localisation of the analysed leaves; being higher in roots and basal leaves, while in the apical leaves it remained at lower levels. These data demonstrate that, unlike the use of the *gus *marker gene, real-time PCR is a powerful tool for detection of chimeras. Although some authors have proposed a consistent, positive Southern analysis as an alternative methodology for monitoring the dissociation of chimeras, our data show that it does not provide enough proof of uniform transformation. In this work, however, real-time PCR was applied successfully to monitor the dissociation of chimeras in tobacco plants and apricot callus.

**Conclusions:**

We have developed a rapid and reliable method to detect and estimate the level of chimeras in transgenic tobacco and apricot plants. This method can be extended to monitor the dissociation of chimeras and the recovery of uniformly-transformed plants.

## Background

Genetic engineering has emerged as a powerful tool to obtain commercial crops with improved agronomic characteristics, and in many plant transformation systems the availability of selectable markers is essential to recover transgenic plants. Genes conferring resistance to selective chemical agents, such as antibiotics and herbicides, are used routinely [1]. However, tissues transformed with these genes and/or with those encoding for desirable traits could harbour both transformed and non-transformed cells. As a result, the regenerated plant will be a chimera for the transgenes. The problem of chimerism seems to be more frequent than originally thought and it has been reported in several herbaceous species, including tobacco [2], soybean [3], potato [4], rice [5], flax [6] and strawberry [7]. Chimeras were also recovered frequently from woody fruit trees such as apple [8]. Very high frequencies have also been reported in *Citrus*, for which escapes and chimeras account for 90% of regenerated lines [9,10].

The occurrence of chimerical plants can be explained most plausibly by the multicellular origin of shoot organogenesis [11,12]. Additionally, the development of chimeras and escapes may result from the transient expression of the marker gene during early stages of the regeneration process or the presence of persistent *Agrobacterium *cells in infected tissues. Chimeras may also be a consequence of the protection of non-transgenic cells by the surrounding transformed cells [10,13] or of the ineffectiveness of selective agents in species with an endogenous tolerance [4].

Because it is extremely important that the transgenes remain stable in time and space throughout the lifetime of the plant, chimeras should be identified and discarded or dissociated, to leave fully-transgenic plants. Several methodologies have been described to monitor the recovery of uniformly-transgenic plants. In combination with the selectable marker gene *npt*II, they are based on the use of reporter genes that allow selection and visual detection of transgene expression, and are used extensively to maximise transformation efficiencies. The most common are based on β-glucuronidase (GUS) staining [14] or green fluorescent protein (GFP) expression [4,15]. However, these methods have serious drawbacks because gene expression can be affected by several factors, including the developmental stage of the plants. Moreover, in many plant species, GFP is hardly detectable in green tissues due to autofluorescence interference [16]. The difficulties found with the use of these visual marker genes led some authors to suggest a consistent, positive Southern as proof of the uniformity of transformation [17,18].

Real-time PCR has emerged as a robust methodology for biological investigations because it can detect and quantify very small amounts of specific nucleic acid sequences. It has been used widely in clinical applications, but recently it has also been reported as a useful tool for estimation of the number of integrations in transgenic plants [19,20], determination of zygosity [21] and quantification of transgene expression [22,23]. Real-time PCR detects PCR products during their accumulation. It is commonly based on either non-specific or specific reporters such as SYBR Green or TaqMan chemistry, respectively [24]. In the former case, a probe labelled with a fluorescent reporter dye at the 5'-end and a quencher at the 3'-end is hybridised to an internal sequence between the forward and reverse PCR primers. When the probe is intact, reporter fluorescence is absorbed via fluorescent resonance energy transfer by the quencher, and no reporter fluorescence can be detected. Due to 5'-exonuclease activity of the *Taq *polymerase, reporter fluorescence accumulates with each successive round of amplification and can be detected. This fluorescent signal is proportional to the amount of PCR product generated and, subsequently, to the initial DNA template in the sample during the exponential phase of PCR, making it suitable for accurate quantification of the starting amounts of nucleic acid in the PCR reaction, without further analyses.

We have developed a new strategy based on the use of quantitative, real-time PCR to allow quantification of the transgene in order to identify chimeras. Moreover, this quantitative method seems to be suitable for monitoring their dissociation.

## Results and discussion

### Validation of the method

**Figure 1:**
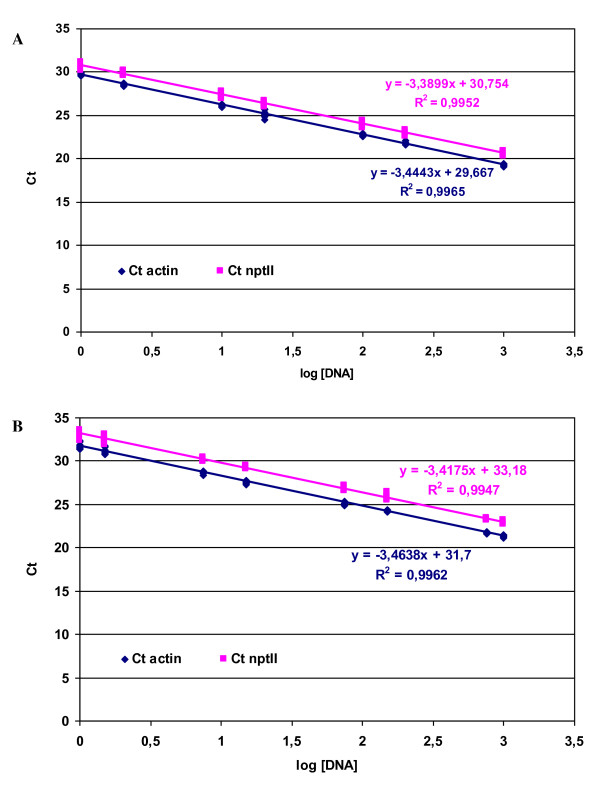
**Efficiency of amplification of *npt *II and *β-actin *genes in three independent DNA extractions from A) apricot callus and B) tobacco leaves, transformed uniformly, with the binary vector pBin19-35SGusintron**. Both apricot callus and tobacco plants harboured one copy of the transgene. DNA samples from transgenic callus and plants were diluted serially to obtain a standard curve for both the endogenous β-*actin *and *npt*II genes and the C_t _value was plotted against log [DNA]. Samples were run in three replicates of each of the three independent DNA extractions. The correlation coefficient and slope values are indicated.

The method used throughout this study is based on the ΔΔC_t _or comparative C_t _method, because it permits higher throughput than the absolute quantification method. We carried out, first, a validation experiment to demonstrate that the reaction efficiencies for the transgene and the internal control are approximately identical [25]. Transgenic apricot and tobacco lines transformed with the binary vector pBin19-35SGusintron, and which carry the *npt*II gene, were obtained and their DNAs were serially diluted to obtain a standard curve for both the endogenous β-*actin *and *npt*II genes. The correlation between the C_t _value and log [DNA] was around 0.99 for *npt*II and *actin *in both apricot and tobacco (Figure 1). This linear relationship indicates that a C_t _value is suitable for estimation of the relative amount of the transgene. In addition, the efficiencies of amplification of the transgene and the internal control were very close (slope = -3.4 for both genes and both plant species, Figure 1) and were not significantly different from a 100% efficiency according to the statistical analysis proposed by Yuan et al. [26] to check quality data and amplification efficiency. For apricot, when regression analyses were run individually for each DNA extraction, amplification values were Rep1: -3.47 and -3.39, Rep2: -3.43 and -3.44, Rep 3: -3.43 and -3.34. For tobacco results were similar and efficiencies for the individual regressions were Rep1: -3.46 and -3.45, Rep2: -3.46 and -3.40 Rep3: -3.47 and -3.43.

All Ct values obtained throughout this work are within the linear range of each target gene.

### Comparison of GUS staining, Southern analysis and real-time PCR for detection of chimeras in tobacco

**Figure 2:**
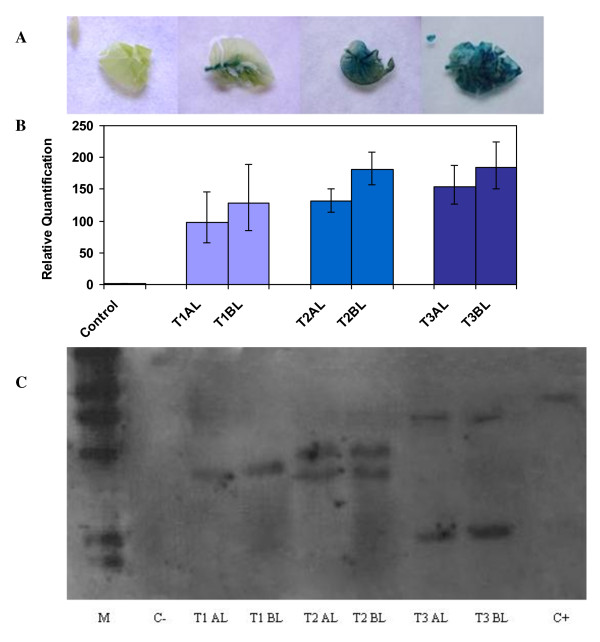
**Detection of chimera in three transgenic lines (from T1 to T3) of tobacco transformed with the binary vector pBin19-35SGusintron, which carry both *npt*II and *gus *genes, using three different methods**. **A) **Histochemical GUS staining of their apical leaves (leaf on the left is a non-transformed control). **B) **Determination of the relative amount of the *npt*II transgene, as compared with a non-transgenic control, in their apical (AL) and basal (BL) leaves using quantitative, real-time PCR. Four independent DNA extraction per line were used. **C) **Southern blot analysis of the *npt*II transgene in the apical (AL) and basal (BL) leaves of the same lines. The lane labelled (+) corresponds to the digested pBin19-35SGusintron vector.

The expression of visual marker genes such as *gus *has been used extensively to detect chimeras in order to discard them [10,17]. However, in apricot plants, *gus *expression seems to be very dependent on the plant developmental stage and, although its expression is uniform and strong in young regenerated buds, it is only expressed in the petioles and veins of leaves from micro-propagated shoots (data not shown). Therefore, it is difficult to use the expression of this gene to discriminate chimerical from uniform plants, since differential expression may be found in different tissues or with plant development. To further demonstrate this point, we used tobacco, a model plant with very-well-established transformation protocols [27]. Several lines transformed with the binary vector pBin19-35SGusintron, which carry both *npt*II and *gus *genes, were obtained and three of them were selected with different degrees of GUS staining in apical leaves of a similar age and developmental stage (Fig 2A). One line (T1) exhibited a weak GUS staining, while the other two (T2 and T3) showed uniform GUS staining. When these lines were evaluated for the relative amount of the *npt*II transgene using quantitative real-time PCR, a positive correlation was found between GUS staining and the amount of *npt*II determined in DNA extracted from the apical or basal leaves. However, the amount of transgene in DNA extracted from apical and basal leaves was significantly different in all lines (P < 0.001 in the three lines), including those in which a uniform GUS staining of the apical leaves was observed (Figure 2B). A consistently higher amount of transgenic DNA was found in basal leaves from all lines, suggesting that the three lines are chimerical.

We also attempted to determine the reliability of Southern blot analysis for monitoring the uniformity of transgenic lines. When the same DNA extractions, used for real-time PCR, were digested with *Bam*HI (with one restriction site into the T-DNA) and subjected to Southern blot analysis using *npt*II as a probe, differences in the pattern of insertion were not observed within the same line, whatever the position of the leaf. However, clear differences in the patterns obtained could be observed among the three lines (Figure 2C). Taken together, these results indicate that regeneration of chimeras did not result only from the fusion of different transformation events, as was reported in *Citrus *[10] and cabbage [17], but rather from the fusion of transgenic and non-transgenic events.

Other authors attempted to dissociate chimerical plants by mean of *in vitro *culture-based methods and used Southern hybridisation to demonstrate that the recovered transgenic tissues were uniform [17,18]. However, our results demonstrate that a positive Southern blot signal will be obtained if the amount of transgene is over the detection threshold, but this does not necessarily mean that all, or even most, of the plant cells are transformed.

### Detection of chimeras in apricot using real-time PCR

**Figure 3:**
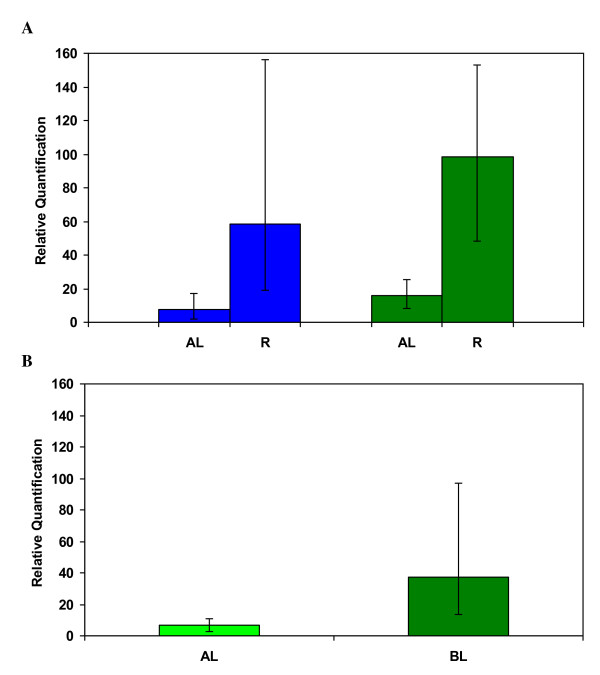
**Detection of chimerism in the transformed apricot lines HN507-31, HPinAB12 and HPinB3**. Plants were transformed with the binary vector pBin19-35SGusintron carrying the *npt*II transgene for kanamycin selection. **A) **DNA was independently extracted from apical (AL) and basal leaves (BL) of five different shoots of the line HN507-31, and used for relative determination of the amount of *npt*II using quantitative, real-time PCR. **B) **DNA was also independently extracted from apical leaves (AL) and from roots (R) of three different shoots of each of the transgenic lines HPinAB12 (blue) and HPinB3 (green), and used for real-time PCR.

Transformation experiments were also carried out with apricot plants using the pBin19-35SGusintron and several transgenic lines were regenerated [15]. Quantification of *npt*II from the apricot line HN507-31, using real-time PCR, revealed that the amounts of the transgene assessed from the DNA extracted from basal and apical leaves of micro-propagated shoots were significantly different (P < 0.01, Figure 3A), indicating that this line was a chimera. In addition, when relative amount of transgene was compared between DNA extractions from roots and apical leaves of several shoots of two different apricot transgenic lines (HPinB3 and HPinAB12), significant differences (P < 0.001 and P < 0.05, respectively) were found between roots and leaves in the relative amount of transgene for both lines (Figure 3B). As with tobacco, the amount of the transgene varied greatly according to the position in the shoot, being very high in roots growing within the selective medium, intermediate in basal leaves and lower in the apical leaves. These results indicate the existence of a gradient for the strength of the antibiotic selection. The antibiotic effect is stronger in those plant parts closer to the selective medium. The chimera is then potentiated as the plant grows and the selection became weaker in distal areas of the plant, located far from the medium.

It is unlikely that this chimeras originated from different events of integration because, in most woody trees, the efficiency of transformation is very low [28].

### Monitoring dissociation of chimera in tobacco and apricot using real-time PCR

**Figure 4:**
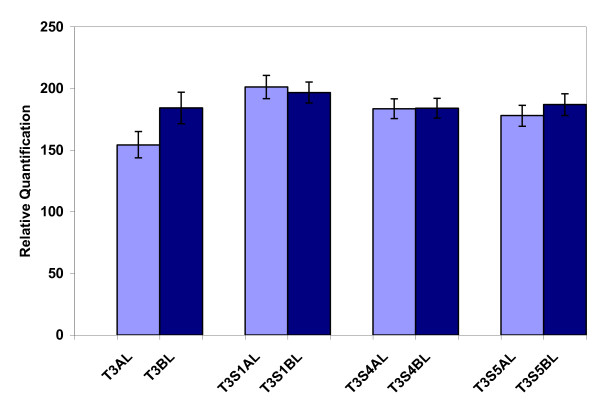
**Dissociation of chimera in the transgenic tobacco line T3**. The chimera was divided in single-node explants and subcultured for 9 additional cycles. Then three different shoots (S1, S4 and S5) were selected because of their good rooting system and dark green leaves and their DNA was extracted from the apical (AL) and basal (BL) leaves. Values are means from four to six independent DNA extractions from each shoot (or eight to twelve from each shoot/position combination).

Transgenic chimerical line T3 in Figure 2 was multiplied *in vitro *by subculturing single-node explants in selective root-inducing medium. Shoots with a good rooting system and dark-green leaves were selected and maintained during 9 subcultures. Then the *npt*II content in apical and basal leaves was evaluated in three different shoots (Figure 4) and there were not significant differences in the *npt*II content between apical and basal leaves. Furthermore, there were neither significant differences between independently selected S4 and S5 shoots, indicating that dissociation of chimera occurred.

**Figure 5:**
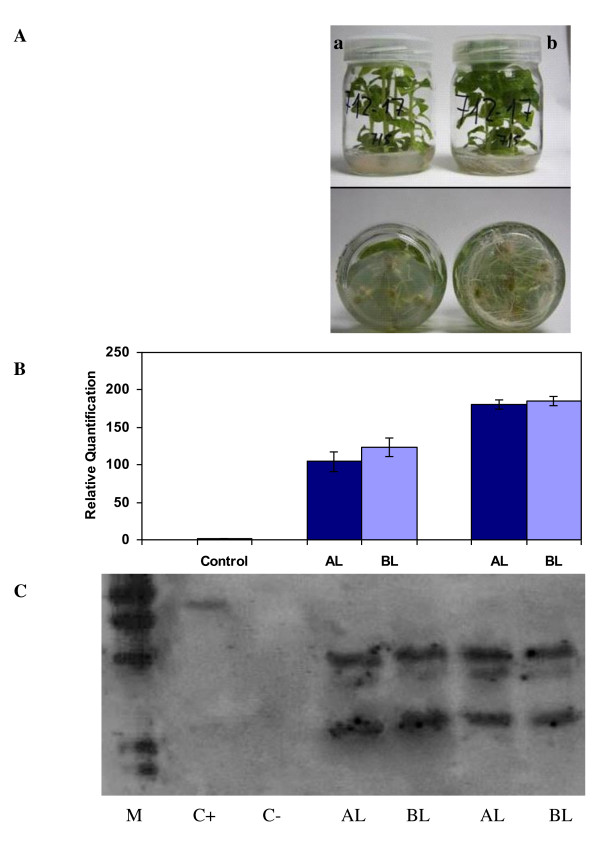
**Dissociation and Quantification of the chimerical tobacco line T17**. **A) **Dissociation of the chimerical tobacco line T17 on rooting medium. Explants were transformed with the binary vector pBin19-35SGusintron carrying the *npt*II transgene for kanamycin selection and screened phenotypically. Shoots (a) in the left-hand jar were chosen for their poor root system, light green leaves and bleaching symptoms, whereas shoots (b) in the right-hand jar were found to be normal. **B) **Quantification of the *npt*II transgene from the apical (AL) and basal leaves (BL) of the shoots a and b using real-time PCR. Values are means from six biological replicates.

We have compared the reliability of Southern blotting and real-time PCR with respect to following the dissociation of chimeras. In tobacco, most chimeras can be dissociated easily by *in vitro *propagation on a selective, root-inducing medium and by phenotypic selection of the best micropropagated shoots (without bleaching symptoms, with a good rooting system, etc.) as shown in Figure 4. To validate this visual observation, chimerical line T17 was selected and shoots were separated, based on a phenotypic classification, into shoots with lower (T17a) and higher (T17b) rooting ability, as well as on the absence/presence, respectively, of bleaching symptoms in the leaves (Figure 5A). Real-time PCR and Southern blot analyses were carried out with DNA extracted from the apical and basal leaves of T17a and T17b shoots. The first method (Figure 5B) confirmed that T17b is transformed uniformly, as the amount of the transgene determined in the apical leaves did not differ significantly from that measured in the basal leaves, unlike T17a for which significant differences (P < 0.05) were found. Moreover, significant differences (P < 0.001) were found in the *npt*II content between T17a and T17b shoots. However, a consistent, positive Southern was observed in both T17a and T17b, whatever the position of the leaf (Figure 5C). This result reinforces the view that this technique is not sufficient to discriminate chimerical from uniformly-transformed plants or to monitor the dissociation of chimeras.

**Figure 6:**
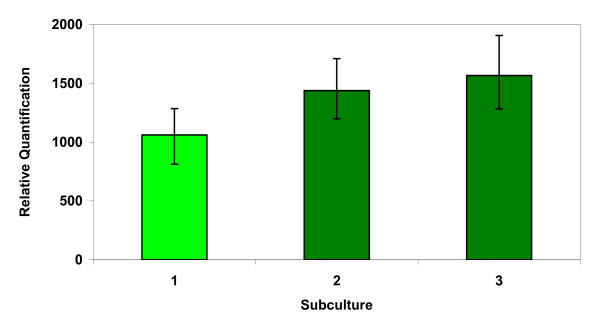
**Dissociation of chimeras in apricot callus**. Explants were transformed initially with the binary vector pBin19-35SGusintron carrying the *npt*II transgene for kanamycin selection. The transgenic line was sub-cultured three times at monthly intervals and its DNA was used for real-time PCR experiments. Values are means from three biological replicates.

Dissociation of chimeras was almost impossible to achieve within the whole apricot plant using *in vitro *micropropagation and an iterative regeneration/selection method [18] may be necessary. We decided to check the validity of real-time PCR for the monitoring of dissociation in transgenic apricot callus tissues. A transgenic callus line, selected randomly, was used to determine the amount of *npt*II after three successive rounds of sub-culture (the period of sub-culture was about one month) (Figure 6). The amount of the transgene did change significantly over this period (P < 0.05), and dissociation occurred after two months (two subculturing cycles) in selective medium. Significant differences were not observed between the last two sub-culture times, but the amount of the transgene significantly differed (P < 0.05) between callus from the third and first subculture. The easy dissociation of chimeras observed in chimerical callus could be explained by the fact that these disorganised tissues are growing in close contact with the selective medium, supporting our assumption that a gradient for the antibiotic effect exists in the entire plant.

## Conclusion

The methodology described here is unique in allowing a quick and easy discrimination of chimerical from uniformly-transformed plants with a very high sensitivity. This methodology is not based on gene expression, which may vary due to a number of different reasons, or to positive/negative results (as in Southern blot analysis) that may depend on the transgene amount being higher than the detection threshold of the technique. It is based on a comparison between relative amounts of transgene in several DNA extractions from the same transgenic line that should be identical in a uniformly transformed plant. Work is in progress to develop an iterative regeneration method that may allow dissociation of chimeras in apricot plants. Also, the factors affecting the initial transformation steps and selection are being optimised, to avoid the production of chimeras.

## Methods

### Plant material, construction and transformation

*Nicotiana tabacum *cv. Xanthi and *Prunus armeniaca *cv. Helena were used as plant material. Shoots of the apricot cv. were micropropagated as described previously [29]. Briefly, they were maintained by sub-culturing at 4-week intervals on a shoot multiplication medium, at 22 ± 1°C under cool white fluorescent tubes (55 μmol m^-2 ^s^-1^), with a 16-h photoperiod. The multiplication medium consisted of QL macronutrients [30] and DKW micronutrients, vitamins and organic compounds [31], 3% sucrose and 0.7% agar (HispanLab, S.A.). The medium was supplemented with 3.1 μM N^6^-benzylaminopurine (BA) and 0.2 μM indole-3-butyric acid, and the pH was adjusted to 5.7 before autoclaving at 121°C for 20 min.

Transformation and regeneration from leaf explants were carried out as described in Petri et al. [15] and both transgenic plants and callus were obtained. The selective medium for callus consisted of QL macronutrients and DKW micronutrients and vitamins supplemented with 3% (w/v) sucrose, 0.7% (w/v) agar, 1 mg L^-1 ^polyvinylpyrrolidone, 0.15% (w/v) hydrolysate of caseine, 0.2 μM BA, 4.52 μM 2,4-dichlorophenoxyacetic acid and 100 mg L^-1 ^kanamycin.

Transgenic tobacco plants were produced according to Horsch *et al. *[27]. They were maintained in MS medium [32] supplemented with 3% (w/v) sucrose, 0.7% (w/v) agar and 75 mg L^-1 ^kanamycin.

For both apricot and tobacco plants, the *A. tumefaciens *strain EHA105, carrying the binary vector pBin19-35SGusintron [33], was used for inoculation of leaf explants. This vector harboured, in its T-DNA, *npt*II for aminoglycoside selection and *gus *for β-glucuronidase histochemical detection. *Npt*II is under the control of the constitutive *Nos *promoter and terminator, whereas *gus *is under the control of the constitutive *35S *promoter and *Nos *terminator.

### DNA extraction and Southern analysis

DNA was extracted from approximately 50 mg of tobacco leaves or apricot leaves and roots, one month after sub-culture, as described by Doyle and Doyle [34]. The youngest, apical or oldest, basal leaves were used.

For Southern analysis, 20 μg of genomic *Bam*HI-digested DNA samples were separated on 1% (w/v) agarose gels and transferred to positively-charged nylon membranes by capillary blotting. The 696-bp PCR *npt*II fragment, amplified using the primers 5'-GATTGAACAAGATGGATTGC-3' and 5'-CCAAGCTCTTCAGCAATATC-3', was labelled with digoxigenin (DIG) using the PCR DIG Probe Synthesis Kit (Roche GmbH, Mannheim, Germany). Pre-hybridisation and hybridisation of filters to labelled probe were performed at 42°C. Blots were then washed twice at 23°C in 2× SSC (0.3 M NaCl, 0.03 M sodium citrate), 0.1% (w/v) sodium dodecyl sulphate (SDS) for 15 min, and twice at 65°C in 0.5× SSC, 0.1% SDS for 15 min. Hybridising bands were visualised with anti-DIG antibody-alkaline phosphatase and CDP-Star (Roche) on X-ray films.

### Histochemical GUS staining

Detection of β-glucuronidase expression was performed on one of the youngest, apical leaves of the tobacco plants, one month after sub-culture, using X-Gluc solution [35]. This consists of 100 mM potassium phosphate pH 8.0, 2 mM Na_2_EDTA, 0.1% Triton X-100, 0.5 mM potassium ferricyanide and ferrocyanide and 0.1% X-Gluc (Duchefa). Leaves were vacuum-infiltrated, incubated overnight at 37°C and chlorophyll was removed using ethanol.

### Primers and probes for real-time PCR reactions

Primers (*npt*II forward 5'-ATCCATCATGGCTGATGCAATGCG-3' and *npt*II reverse 5'-GATGTTTCGCTTGGTGGTCGAAT-3'; apricot β-*actin *forward 5'-TGCCTGCCATGTAT GTTGCCATCC-3' and β-*actin *reverse 5'-AACAGCAAGGTCAGACGAAGGAT-3'; tobacco β-*actin *forward 5'-CTGGCATTGCAGATCGTATGA-3' and β-*actin *reverse 5'-GCGCCACCACCTTGATCTT-3') and probes (*npt*II 5'-TGCATACGCTTGATCCGGCTAC CT-3'; apricot β-*actin *5'-TGATGGTGTGAGTCACACTGTGCCAA-3'; and tobacco β-*actin *5'-AAGGAAATTACTGCTCTTGC-3') were designed on the basis of published sequences in the GenBank database, by using Primer Express software (Applied Biosystem, CA), to amplify an 80-bp amplicon of the *npt*II, an 140-bp amplicon of the apricot *actin *and 75-bp of the tobacco *actin*. The probe for *npt*II was labelled at the 5'-end with VIC as a reporter and with 6-FAM for tobacco and apricot *actin*. Both probes used MGB-NFQ as a quencher at their 3'-end. The PCR reactions consisted of 1× TaqMan Universal Master Mix (Applied Biosystem, CA). This contains an AmpliTaq Gold DNA polymerase, AmpErase uracyl-N-glycosylase to prevent contamination from previous PCR reactions, dNTPs, a passive dye reference (ROX), 100 nM of each specific probe, 600 nM of the specific primers (for the transgene and the internal control) and 2 μl of DNA. The reaction mixtures were made up to a final volume of 25 μl with water. Reactions were performed in 96-well reaction plates, and monitored with an ABI-PRISM 7500 Sequence Detection System (Applied Biosystem, CA). The PCR reactions for *npt*II and *actin *were run simultaneously in the same well plate. The cycling parameters were as follows: one cycle at 50°C for 2 min, one cycle at 95°C for 10 min and 40 cycles of 95°C for 15 sec (denaturation) and 60°C for 1 min (annealing and extension).

### Estimation of the relative amount of the transgene

For calculation of relative transgene content, we used the 2^-ΔΔCt ^method [22]. The Ct value was adjusted automatically and the threshold cycle value difference (ΔC_t_) between VICC_t _of the target gene (*npt*II) and FAMC_t _of *βactin *(internal control) was used to normalise the amount of *npt*II transgene. As long as the target gene and the internal control have similar amplification efficiencies, C_t _values are normalised by using the difference (ΔC_t_) between the internal control and target gene. This value is calculated for each sample to be quantified. Because the *β-actin *is constant relative to the total genomic DNA, the ΔC_t _between the *npt*II transgene and the *β-actin *gene in each sample corresponds to the normalisation of the Ct value for the *npt*II. Finally, the relative quantification of the *npt*II transgene in each sample was calculated according to the formula where the reference sample was a non-transformed control:

$$\begin{array}{l} \text{Relative Quantification}=2^{-\Delta \Delta Ct}\\ \text{Where }\Delta \Delta {\text{C}}_{\text{t}}=\Delta {\text{C}}_{\text{t}}(\text{unknown sample})-\Delta {\text{C}}_{\text{t}}(\text{reference sample}). \end{array}$$

### Experimental design and data analysis

The hypothesis sustaining this manuscript is that relative transgene content within a specific uniformly transformed plant or line should remain constant throughout all tissues of the plant, different shoots of one line and at different times. Therefore, there should not be significant differences when comparing the relative amount of transgene between apical and basal leaves of one transgenic shoot, different shoots or different subcultures of one transgenic line. To test uniformity of transformation, qPCR were run with, at least, three biological replicates (independent DNA extractions) from each position in the shoot and/or each shoot of a transgenic line. Each PCR was run, at least, in three technical replicates from each independent DNA extraction. Relative amount of transgene was calculated for each replicate.

A linear mixed model was fitted to the normalised Ct (ΔΔCt) where shoots and/or position in the plant and/or subculture time are considered fixed factorial effects, and biological replicates a random effect within each shoot-position combination. When a fixed factor was significant (P < 0.05) the transgenic line was classified as a chimera. Mean relative quantification and 95% confidence intervals are shown in all figures for easier interpretation of results.

For the validation experiment, 7 dilutions were performed for each of three biological replicates (three independent DNA extractions), from uniformly-transformed tobacco and apricot tissues with only one copy of the transgene, and reactions were run in three replicates from each dilution.

## Authors' contributions

MF and LF were involved in the transformation experiments, performed histochemical GUS staining and carried out real-time PCR, Southern blot analyses and data analyses. LB conceived the idea of using real-time PCR to discriminate chimeras, performed transformation experiments and was responsible for the coordination of the study and statistical analyses. All the authors participated in the design of the experiments and approved this version of the manuscript.

## References

[B1] Darbani B Eimanifar A Stewart CN Camargo WN Methods to produce marker-free transgenic plants Biotechnology Journal 2007 2 83 90 10.1002/biot.200600182 17167767

[B2] Schmülling T Schell J Transgenic tobacco plants regenerated from leaf disks can be periclinal chimeras Plant Mol Biol 1993 21 705 708 10.1007/BF00014554 8448369

[B3] Christou P Morphological description of transgenic soybean chimeras created by the delivery, integration and expression of foreign DNA using electric discharge particle acceleration Ann Bot 1990 66 379 386

[B4] Rakosy-Tican E Aurori CM Dijkstra C Thieme R Aurori A Davey MR The usefulness of the *gfp *reporter gene for monitoring *Agrobacterium*-mediated transformation of potato dihaploid and tetraploid genotypes Plant Cell Rep 2007 26 661 671 10.1007/s00299-006-0273-8 17165042

[B5] Christou P Ford TL Recovery of Chimeric Rice Plants from Dry Seed using Electric Discharge Particle Acceleration Ann Bot 1995 75 449 454 10.1006/anbo.1995.1044

[B6] Dong JZ McHughen A Transgenic flax plants from *Agrobacterium *mediated transformation: incidence of chimeric regenerants and inheritance of transgenic plants Plant Sci 1993 91 139 148 10.1016/0168-9452(93)90137-O

[B7] Mathews H Wagoner W Kellogg J Bestwick RK Genetic transformation of strawberry: Stable integration of a gene to control biosynthesis of ethylene In Vitro Cell Dev Biol -Plant 1995 31 36 43 10.1007/BF02632224

[B8] Flachowsky H Riedel M Reim S Hanke V Evaluation of the uniformity and stability of T-DNA integration and gene expression in transgenic apple plants Electronic Journal of Biotechnology 2008 11 1 15 10.2225/vol11-issue1-fulltext-10

[B9] Costa MGC Otoni WC Moore GA An evaluation of factors affecting the efficiency of *Agrobacterium*-mediated transformation of *Citrus paradisi *(Macf.) and production of transgenic plants containing carotenoid biosynthetic genes Plant Cell Rep 2002 21 365 373 10.1007/s00299-002-0533-1

[B10] Domínguez A Cervera M Pérez RM Romero J Fagoaga C Cubero J Characterisation of regenerants obtained under selective conditions after *Agrobacterium*-mediated transformation of citrus explants reveals production of silenced and chimeric plants at unexpected high frequencies Mol Breeding 2004 14 171 183 10.1023/B:MOLB.0000038005.73265.61

[B11] Zhu XY Zhao M Ma S Ge YM Zhang MF Chen LP Induction and origin of adventitious shoots from chimeras of *Brassica juncea *and *Brassica oleracea* Plant Cell Rep 2007 26 1727 1732 10.1007/s00299-007-0398-4 17622536

[B12] Poethig S Genetic mosaics and cell lineage analysis in plants Trends in Genetics 1989 5 273 277 10.1016/0168-9525(89)90101-7 2686117

[B13] Park SH Rose SC Zapata C Srivatanakul M Smith RH Cross-protection and selectable marker genes in plant transformation In Vitro Cell Dev Biol-Plant 1998 34 117 121 10.1007/BF02822775

[B14] McHughen A Jordan MC Recovery of transgenic plants from "escape" shoots Plant Cell Rep 1989 7 611 614 10.1007/BF00272041 24240442

[B15] Petri C Wang H Alburquerque N Faize M Burgos L *Agrobacterium*-mediated transformation of apricot (*Prunus armeniaca *L.) leaf explants Plant Cell Rep 2008 27 1317 1324 10.1007/s00299-008-0550-9 18449544

[B16] Billinton N Knight AW Seeing the wood through the trees: A review of techniques for distinguishing green fluorescent protein from endogenous autofluorescence Analytical Biochemistry 2001 291 175 197 10.1006/abio.2000.5006 11401292

[B17] Berthomieu P Béclin C Charlot F Doré C Jouanin L Routine transformation of rapid cycling cabbage (Brassica oleracea). Molecular evidence for regeneration of chimeras Plant Sci 1994 96 223 235 10.1016/0168-9452(94)90240-2

[B18] Mathews H Dewey V Wagoner W Bestwick RK Molecular and cellular evidence of chimaeric tissues in primary transgenics and elimination of chimaerism through improved selection protocols Transgenic Res 1998 7 123 129 10.1023/A:1008872425917

[B19] Mason G Provero P Vaira AM Accotto GP Estimating the number of integrations in transformed plants by quantitative real-time PCR BMC Biotechnology 2002 2 20 10.1186/1472-6750-2-20 12398792 PMC137580

[B20] Yi CX Zhang J Chan KM Liu XK Hong X Quantitative real-time PCR assay to detect transgene copy number in cotton (*Gossypium hirsutum*) Analytical Biochemistry 2008 375 150 152 10.1016/j.ab.2007.11.022 18078801

[B21] Schmidt MA Parrott WA Quantitative detection of transgene in soybean (*Glycine max *L., Merrill) and peanut (*Arachis hypogaea *L.) by real-time polymerase chain reaction Plant Cell Rep 2001 20 422 428 10.1007/s002990100326 24549450

[B22] Livak KJ Schmittgen D Analysis of relative gene expression data using real-time quantitative PCR and the 2^-^^Ct^_method_ Methods 2001 25 402 408 10.1006/meth.2001.1262 11846609

[B23] Shih SC Smith LEH Quantitative multi-gene transcriptional profiling using real-time PCR with a master template Experimental and Molecular Pathology 2005 79 14 21 10.1016/j.yexmp.2005.03.004 15894312

[B24] Heid CA Stevens J Livak KJ Williams PM Real time quantitative PCR Genome Res 1996 6 986 994 10.1101/gr.6.10.986 8908518

[B25] Ingham DJ Beer S Money S Hansen G Quantitative Real-Time PCR Assay for Determining Transgene Copy Number in Transformed Plants BioTechniques 2001 31 132 141 11464506 10.2144/01311rr04

[B26] Yuan JS Burris J Stewart NR Mentewab A Stewart CN Jr Statistical tools for transgene copy number estimation based on real-time PCR BMC Bioinformatics 2007 8 S6 10.1186/1471-2105-8-S7-S6 18047729 PMC2099498

[B27] Horsch RB Fry JE Hoffmann NL Eichholtz D Rogers SG Fraley RT A simple and general method for transferring genes into plants Science 1985 227 1229 1231 10.1126/science.227.4691.1229 17757866

[B28] Petri C Burgos L Transformation of fruit trees. Useful breeding tool or continued future prospect? Transgenic Res 2005 14 15 26 10.1007/s11248-004-2770-2 15865045

[B29] Pérez-Tornero O Burgos L Different media requirements for micropropagation of apricot cultivars Plant Cell, Tiss Org Cult 2000 63 133 141 10.1023/A:1006430718024

[B30] Quoirin M Lepoivre P Etude de milieux adaptes aux cultures *in vitro *de *Prunus* Acta Hort 1977 78 437 442

[B31] Driver JA Kuniyuki AH *In vitro *propagation of Paradox walnut rootstock HortScience 1984 19 507 509

[B32] Murashige T Skoog F A revised medium for rapid growth and bio assays with tobacco tissue cultures Physiol Plant 1962 15 473 497 10.1111/j.1399-3054.1962.tb08052.x

[B33] Vancanneyt G Schmidt R O'Connor-Sanchez A Willmitzer L Rocha-Sosa M Construction of an intron-containing marker gene: Splicing of the intron in transgenic plants and its use in monitoring early events in *Agrobacterium*-mediated plant transformation Mol Gen Genet 1990 220 245 250 10.1007/BF00260489 2325623

[B34] Doyle JJ Doyle JL Isolation of DNA from fresh tissue Focus 1990 1990 13 15

[B35] Jefferson RA Assaying chimeric genes in plants: The GUS gene fusion system Plant Mol Biol Rep 1987 5 387 405 10.1007/BF02667740

